# Assessment of the Effects of Single-Domain Anti-Idiotypic Distribution Enhancers on the Disposition of Trastuzumab and on the Efficacy of a PE24-Trastuzumab Immunotoxin

**DOI:** 10.3390/cancers17091468

**Published:** 2025-04-27

**Authors:** Ping Chen, Yu Zhang, Brandon M. Bordeau, Joseph P. Balthasar

**Affiliations:** Department of Pharmaceutical Sciences, University at Buffalo, Buffalo, NY 14214, USA; pchen39@buffalo.edu (P.C.); zhang238@buffalo.edu (Y.Z.); bmbordea@buffalo.edu (B.M.B.)

**Keywords:** binding-site barrier, anti-idiotypic distribution enhancers (AIDEs), immunotoxin, trastuzumab, PE24, pharmacokinetics/pharmacodynamics (PK/PD) modeling, antibody tumor distribution, antibody selectivity

## Abstract

Background/Objectives: Antibody-based therapies often exhibit limited distribution within solid tumors due to the “binding-site barrier” (BSB). Our group has developed and validated the use of anti-idiotypic distribution enhancers (AIDEs), which transiently block antibody binding, improving intra-tumoral distribution and efficacy. This study evaluated 1HE and LG1, model anti-trastuzumab AIDEs, in combination with trastuzumab–PE24, a highly potent immunotoxin. Methods: The effects of 1HE on the whole-body disposition of radiolabeled trastuzumab were assessed in NCI-N87 tumor-bearing mice. Mechanistic pharmacokinetic/pharmacodynamic (PK/PD) modeling was employed to explore how AIDE binding kinetics influence antibody intra-tumoral distribution and immunotoxin potency. Trastuzumab–PE24 was developed by site-specific conjugation, enabled by self-splicing split intein, with cytotoxicity tested on various cell lines in vitro. The impact of 1HE and LG1 coadministration on trastuzumab–PE24 efficacy was evaluated in NCI-N87 xenograft-bearing mice. Results: 1HE coadministration decreased trastuzumab tumor maximum concentration, reducing tumor terminal slope by 8% and overall tumor exposure by 2.6%, without negatively affecting selectivity. Modeling predicted the optimal AIDE dissociation rate constant for trastuzumab–PE24 to be between 0.015 and 0.3 h^−1^. The coadministration of trastuzumab–PE24 with 1HE and LG1 improved anti-tumor efficacy and extended median survival to 60 days (*p* = 0.0002). Conclusions: AIDE coadministration led to minimal negative impacts on overall tumor exposure, consistent with model simulations. AIDE coadministration improved the efficacy of trastuzumab–PE24 in NCI-N87 xenografts. Modeling further predicted that repeated AIDE administration with trastuzumab–PE24 could induce complete tumor regression. These findings highlight the advantages of the AIDE strategy, particularly when coadministered with highly potent immunotoxins.

## Simple Summary

The efficacy of antibody-based therapies applied to solid tumors is often limited by the “binding-site barrier” (BSB). Our group has developed anti-idiotypic distribution enhancers (AIDEs), which enhance antibody intra-tumoral distribution and efficacy. This study evaluates 1HE and LG1, model anti-trastuzumab AIDEs, and trastuzumab–PE24, a site-specific conjugated immunotoxin with high potency. Mechanistic pharmacokinetic/pharmacodynamic (PK/PD) modeling was used to investigate relationships between AIDE binding characteristics and effects on antibody distribution and immunotoxin potency. AIDE coadministration led to negligible negative impacts on overall exposure and pharmacokinetic selectivity. Consistent with prior work demonstrating that AIDEs improve intra-tumoral antibody distribution and anti-cancer efficacy, 1HE and LG1 enhanced the anti-tumor efficacy of trastuzumab-PE in NCI-N87 xenografts. In addition, the PK/PD model predicted that the repeated administration of AIDEs with trastuzumab–PE24 could lead to complete tumor regression. This model prediction implies that the benefits of the AIDE approach increase with the increasing potency of immunotoxins.

## 1. Introduction

The poor uptake and distribution of anti-cancer antibodies within tumors are longstanding problems in oncology, attributed to various factors including vasculature barriers, matrix barriers, and cellular or antigen barriers [1]. Among these, the binding-site barrier (BSB) presents a significant obstacle. The BSB describes the tendency of high-affinity antibodies to bind to tumor antigens near extravasation sites, thereby hindering their penetration into deeper regions of solid tumors. This results in heterogeneous tumor distribution and sub-optimal therapeutic efficacy [2,3,4].

To mitigate these issues, we recently introduced a strategy to enhance the intra-tumoral distribution of antibodies and antibody conjugates: the transient, competitive inhibition of antibody binding to tumor antigens facilitated by co-administered anti-idiotypic single domain antibodies (sdAb), referred to as anti-idiotypic distribution enhancers (AIDEs). In this approach, AIDEs bind with high affinity to the antigen-binding sites in the variable domains of anti-tumor monoclonal antibodies (mAbs). When AIDEs block the binding between the antibody and tumor antigens, the resulting antibody–AIDE complexes can diffuse into deeper regions within the tumor. Following the dissociation of the AIDE from the antibody, the antibody can bind to the tumor antigens, thereby achieving a more homogeneous tumor distribution. We hypothesize that this transient inhibition of antibody binding by AIDEs can promote the increased penetration of antibodies within the tumor, without sacrificing the advantages of high-affinity binding to tumor antigens. Implementing the AIDE approach resulted in an improved intra-tumoral distribution of trastuzumab and trastuzumab–gelonin [5,6].

We have previously shown that the anti-cancer efficacy of trastuzumab-DM1 and trastuzumab–gelonin in NCI-N87 tumor-bearing mice was significantly improved when coadministered with AIDEs [5,6]. Additionally, the administration of 1HE, an anti-trastuzumab AIDE, was previously found not to affect the plasma pharmacokinetics (area under the concentration curve (AUC)_0–10 days_) of trastuzumab at doses of 0.1, 1, and 10 mg/kg, nor of T-DM1 at the dose of 1.8 mg/kg in non-tumor-bearing mice [5]. However, the effect of AIDE coadministration on tumor exposure and pharmacokinetic selectivity was previously unexplored.

Based on modeling and simulation, we hypothesize the utility of AIDE approach will be most pronounced when applied to antibodies delivering highly potent toxins that have steep concentration–effect relationships. In contrast to gelonin, which exhibits an inefficient endosomal escape mechanism and poor intracellular toxicity [7], Pseudomonas Exotoxin (PE) possesses both toxin and translocation domains, which could facilitate the cytosolic process autonomously [8,9]. This mechanism of cellular intoxication by PE has led to high response rates, but it incurs severe adverse events [10,11]. For instance, Lumoxiti, an immunotoxin composed of an anti-CD22 disulfide-stabilized variable fragment (dsFv) and PE38, received FDA approval in 2018, but was accompanied by a “black-box” warning of capillary leak syndrome, liver toxicity and kidney toxicity [12,13]. Lumoxiti also demonstrated high immunogenicity in patients, and was subsequently withdrawn in 2023 [14]. Additionally, there is currently no immunotoxin approved for the treatment of solid tumors; all approved immunotoxins are targeting hematological cancers [10]. Thus, the issues of immunogenicity, adverse events, and solid tumor targeting continue to hamper the development of PE toxins.

Several PE38 derivatives have been designed to reduce immunogenicity. Among these, PE24, with domain II deleted but preserving the furin cleavage site, exhibits substantially reduced immunogenicity while retaining high potency [15,16]. PE24-LO10R was engineered through mutation to minimize B-cell epitopes, which further decreased its immunogenicity [17]. Currently, LMB-100, employing this mutated PE24, is in clinical trials for patients with advanced solid tumors expressing mesothelin. Phase I studies revealed that while it was less immunogenic than Lumoxiti, it displayed only limited anti-tumor efficacy for solid tumor treatment, and dose-limiting toxicity was noted [18]. For these PE-derived immunotoxins, the toxicity of the conjugated payloads may limit maximum tolerated immunotoxin doses to values below those required for tumor regression. We propose that the AIDE approach may substantially enhance the efficacy and clinical utility of these highly potent conjugates. This work evaluated the impact of model AIDEs, 1HE and LG1, on antibody pharmacokinetics, tumor distribution, and on the anti-tumor efficacy of trastuzumab–PE24.

## 2. Materials and Methods

### 2.1. Cell Lines

Ramos (CRL-1596, RRID: CVCL_0597) and SKBR3 cells (HTB-30, RRID: CVCL_0033) were purchased from ATCC (Manassas, VA) and were cultured following ATCC recommendations. NCI-N87 (CRL-5822, RRID: CVCL_ 1603) were a generous gift from Dr. Dhaval Shah.

### 2.2. Pharmacokinetics of Trastuzumab in NCI-N87 Tumor-Bearing Mice

Athymic nude mice aged 4 weeks were purchased from the Jackson Laboratory and were injected subcutaneously into the right flank with 5 million NCI-N87 cells/mouse. Trastuzumab was radiolabeled with ^125^I with the modified chloramine-T method as described previously [19]. Two days before dosing, the mouse drinking water was replaced with autoclaved 0.2 g/L KI in order to avoid the uptake of free ^125^I. Once tumors reached 200–300 mm^3^, 2.5 mg/kg trastuzumab with respective 400 μCi ^125^I-labeled tracer doses, with or without 1HE at a 4:1 molar ratio, was administered intravenously. Mice were terminally sacrificed at 3 h and 8 h, and at 1, 3, 7, and 10 days (*n* = 3/time point), where blood and tissues were harvested. Blood samples were centrifuged to collect plasma. Then, plasma samples were precipitated by TCA (Sigma Life Sciences, St. Louis, MO, USA) to remove free ^125^I. Briefly, 20 μL plasma for each sample was precipitated on ice for 15 min with 200 μL of 1% *v*/*v* BSA and 700 μL 10% *w*/*v* TCA in PBS. Then, samples were centrifuged for five minutes to pellet the precipitates. After three rounds of washing with PBS, the TCA-precipitated plasma samples were assessed for gamma radiation. The harvested tissues were weighed and counted by a gamma counter. Plasma pharmacokinetics and tissue pharmacokinetics were calculated using noncompartmental analysis (NCA) in Phoenix^®^ WinNonLin 7 (Phoenix, Pharsight Corporation, Palo Alto, CA, USA).

### 2.3. Preparation of Trastuzumab–PE24

N-terminal intein was genetically fused to the C-terminus of the heavy chain of trastuzumab. The plasmid DNA of the heavy chain and the light chain in pcDNA3.4 were synthesized by GeneArt (Thermo Fisher Scientific, Waltham, MA, USA) and transformed to E.Coli strain NEB-5alpha (New England BioLabs, Ipswich, MA, USA). Large-scale plasmids were prepared by growing a 250 mL overnight culture and purified using ZymoPURE II plasmid maxiprep kit (Zymo Research, Irvine, CA, USA). The plasmids were then transfected into ExpiCHO-S cells (Thermofisher, Grand Island, NY, USA) and produced for 10 days post-transfection. Supernatants were collected by centrifugation at 10,000× *g* for 15 min and then filtered through the 0.22 μm filter-top. Antibodies were purified by a HiTrap Protein G Chromatograph (GE Healthcare Life Sciences, Uppsala, Sweden). C-terminal intein was fused to the N terminus of the toxin PE24, and the gene coding the Intein–PE24 was optimized and synthesized by GeneArt (Thermo Fisher Scientific, Waltham, MA, USA), with the NdeI restriction site at the N terminus and NheI restriction site at the C terminus. Genes were restriction-digested and ligated into pET-22b (Millipore-Sigma, Burlington, MA, USA, 69744). Then intein–PE24 was expressed in the E. coli strain Shuffle T7 (New England Biolabs, Ipswich, MA, USA, C3029J). Once the OD 600 was 0.6~0.8, cells containing plasmids were induced with IPTG for 18–22 h at 16 °C. Intein–PE24 was harvested via centrifugation at 10,000× *g* for 5 min, lysed by Bugbuster lysis buffer (MilliporeSigma, Burlington, MA, USA) and purified using a HisPur^TM^ Ni-NTA Spin Column (Thermo Fisher Scientific, Waltham, MA, USA, 88226).

For intein self-splicing conjugation, trastuzumab–intein was bound to the protein A resin in the equilibrium buffer. After washing, Intein–PE24 at either 8 molar excess or 20 molar excess and TCEP (Thermo Fisher Scientific, Waltham, MA, USA) were added onto the column. The reaction was conducted for 24 h at room temperature under shaking. After washing 3 times, 100 molar equivalent dehydroascorbic acid in PBS was added and incubated for 2 h at 37 °C under shaking. The conjugates were then desalted into nickel equilibrium buffer and purified using the HisPur^TM^ Ni-NTA resin (Thermo Fisher Scientific, Waltham, MA, USA) to remove unconjugated trastuzumab. The purified conjugates were analyzed by SDS-PAGE under reducing and non-reducing conditions to evaluate the toxin–antibody ratio (TAR) [20].

### 2.4. Cell Viability Assay

Log-phase HER2-expressing cell lines SKBR3 cells, NCI-N87 cells or RAMOS cells were seeded in a 96-well plate at a density of 5000 cells/well. After 24 h, culture media was replaced with 200 μL of various concentrations of trastuzumab–PE24, trastuzumab–intein or intein–PE24. After a 6-day incubation, 100 μL of complete media and 25 μL of 5 mg/mL of 3-(4,5-dimethylthiazol-2-yl)-2,5-diphenyltetrazolium bromide (MTT) (Sigma, St. Louis, MO, USA) solution were added and incubated for 4 h. Then, 100 μL of 10% SDS in 0.01 M HCl was added to solubilize the formazan crystals. The plates were read at 590 nm and 640 nm (Spectromax, Molecular Devices, Sunnyvale, CA, USA). Half-maximal inhibitory concentration values (IC50) were determined by fitting a cell growth inhibition model with variable slope using GraphPad Prism 7 (GraphPad Software, Inc., San Diego, CA, USA).

### 2.5. Pharmacokinetic/Pharmacodynamic Modeling and Simulation

The sphere pharmacokinetic (PK) model has been described previously [6,21]. The pharmacodynamic (PD) model defines three tumor cell states—dividing cells, intoxicated cells, and dead cells. Once the trastuzumab–PE24 is internalized, the PE toxins translocate from endosomes to the cytosol. The intoxication rate is governed by the number of toxin molecules accumulated in the cell cytosol in each tumor cell. Then, protein synthesis stops in the dead cell, and the dead cells are removed from the tissue. PD parameters, including endosomal degradation rate constant (ke), cytosolic degradation rate constant (kc), cytosolic translocation rate constant (kt), half-maximal killing concentration values (TC50), maximal intoxication rate constant (K_cat_^max^), and dead cell removal rate constant (kcl), can be found in the work of Pak et al. [22,23]. The simulations were conducted in Berkeley Madonna.

### 2.6. Trastuzumab–PE24 Efficacy Study

Male and female nude mice were purchased from the Jackson Laboratory and were injected subcutaneously with 5 million NCI-N87 cells/mouse, as described above. Once the tumor volume of NCI-N87 xenograft mice reached 100 mm^3^, mice were intravenously injected via the retro-orbital sinus plexus with PBS, 0.15 mg/kg trastuzumab-PE, or trastuzumab-PE with LE8 or 1HE administered at a ten-fold molar excess. Mice body weights and tumor sizes were monitored every other day. Tumor volumes were calculated using TV = 0.5 × l^2^ × w, where l represents the longest diameter of the tumor and w represents the diameter perpendicular to l. Mice were sacrificed if the tumor diameter exceeded 20 mm or weight loss was larger than 15%. The log-rank test using GraphPad Prism 7 (GraphPad Software, Inc., San Diego, CA, USA) was used to evaluate the Kaplan–Meier survival curves.

## 3. Results

### 3.1. Effects of 1HE on Trastuzumab Tumor Exposure and Pharmacokinetic Selectivity

**Figure 1:**
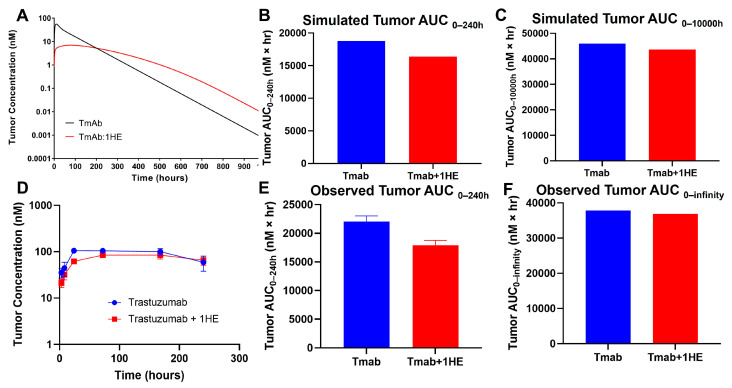
Simulated and observed trastuzumab tumor pharmacokinetics. (**A**) Sphere model-simulated tumor pharmacokinetics, (**B**) tumor AUC_0–240h_ or (**C**) tumor AUC_0-infinity_ of trastuzumab alone (blue) or trastuzumab with the coadministration of 1HE (red). (**D**) Observed tumor pharmacokinetics, (**E**) tumor AUC_0–240h_ or (**F**) tumor AUC_0-infinity_ of trastuzumab alone (blue) or trastuzumab with the coadministration of 1HE (red) following an intravenous 2.5 mg/kg dose in NCI-N87 tumor-bearing mice (*n* = 3/time point).

Mechanistic mathematical modeling was employed to estimate the impact of 1HE on trastuzumab tumor exposure. Predictive simulations suggested that the coadministration of 1HE could reduce the tumor maximal concentration (Cmax) of trastuzumab and decrease the tumor exposure (AUC_0–240 h_) by 20% (Figure 1A,B). However, beyond approximately 200 h, the tumor concentrations in mice coadministered with 1HE were predicted to exceed control values. Consequently, the tumor AUC_0-infinity_ for trastuzumab was anticipated to be comparable (Figure 1C).

**Figure 2:**
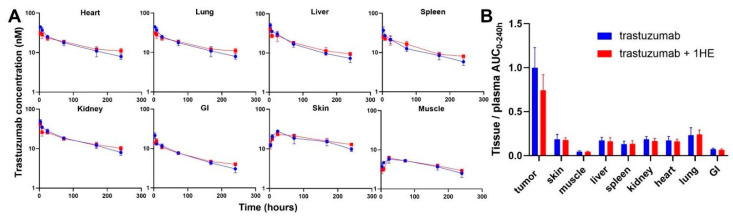
Observed tissue pharmacokinetics and pharmacokinetic selectivity from 0 to 240 h. (**A**) Tissue pharmacokinetics of trastuzumab alone (blue) or trastuzumab with the coadministration of 1HE (red) following an intravenous 2.5 mg/kg dose in NCI-N87 tumor-bearing mice (*n* = 3/time point). (**B**) Comparison of tissue selectivity for trastuzumab alone (blue) or trastuzumab+1HE (red) based on the calculation of tissue:plasma AUC ratios.

To experimentally validate the simulation results of the effect of 1HE on trastuzumab pharmacokinetics, radio-labeled trastuzumab was intravenously administered to NCI-N87 tumor-bearing mice either with or without 1HE. Samples from plasma, tumor, skin, muscle, liver, spleen, kidney, heart, lung, and the gastrointestinal tract were collected at specified time points. Radioactivity and trastuzumab concentrations were determined using a gamma counter. In accordance with the simulated results, 1HE coadministered with trastuzumab displayed reduced tumor Cmax and a decrease in tumor AUC up to 10 days (Figure 1D,E). Beyond 240 h, however, the tumor concentration of mice treated with 1HE exceeded that of the control group, and the tumor terminal slope decreased by 8%. Thus, the tumor AUC_0-infinity_ with or without 1HE coadministration showed a negligible change, exhibiting a mere 2.6% difference (Figure 1F). Besides tumor, AIDE coadministration had no impact on the pharmacokinetic selectivity of trastuzumab, as the tissue AUC relative to plasma AUC from 0 to 240 h remained unaltered (Figure 2A,B).

### 3.2. Predicted Relationship Between Immunotoxin Potency and the Benefits of the AIDE Approach

**Figure 3:**
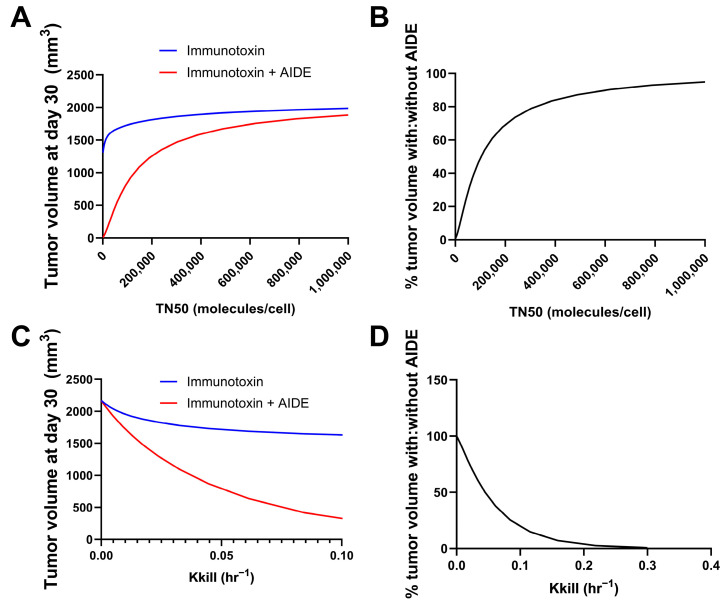
Predicted relationship between immunotoxin potency and the benefits of the AIDE approach. Simulations of tumor volumes were performed using the previously developed trastuzumab–gelonin model. (**A**) Shown is the tumor volume on day 30 following the single administration of immunotoxin with different TN50s, with (red) or without (blue) 1HE. (**B**) The ratio of the tumor volume of immunotoxins with AIDE to without AIDE was calculated and is shown as a percentage. (**C**) Shown is the tumor volume on day 30 following the single administration of an immunotoxin with different Kkill, with (red) or without (blue) 1HE. (**D**) The ratio of the tumor volume of immunotoxins with AIDE to without AIDE was calculated and is shown as a percentage.

Simulations were performed using the previously developed trastuzumab–gelonin model [6]. We consider the binding events in the tumor microenvironment to be a result of multiple factors; most predominantly, the intrinsic binding kinetics and diffusion/convection of the molecules. As such, the model incorporates the association, dissociation and diffusion of antibodies and AIDEs in the sphere of the tumor model for describing the binding kinetics in the tumor microenvironment. Model parameters were all obtained from the literature. The potency of the immunotoxins was changed by changing the maximum kill rate constant (Kkill) or the internalized immunotoxins/cell for 50% of Kkill (TN50). The tumor volumes on day 30 following the single administration of immunotoxin, with or without 1HE, were simulated. A smaller TN50 indicated a higher potency of immunotoxins, which was predicted to correlate with more reduced tumor volume at day 30 using the AIDE approach (Figure 3A). The ratio of tumor volume with AIDE to without AIDE was calculated and is shown in Figure 3B. Smaller TN50 demonstrated more pronounced decreases in tumor volumes, underscoring the greater benefits of the AIDE approach. Similarly, larger Kkill values (indicative of higher potency) were predicted to allow greater reductions in tumor volumes (Figure 3C,D), further supporting the better utility of the AIDE approach against the more potent immunotoxins.

### 3.3. Conjugation and Cytotoxicity of Trastuzumab–PE24 Immunoconjugates

**Figure 4:**
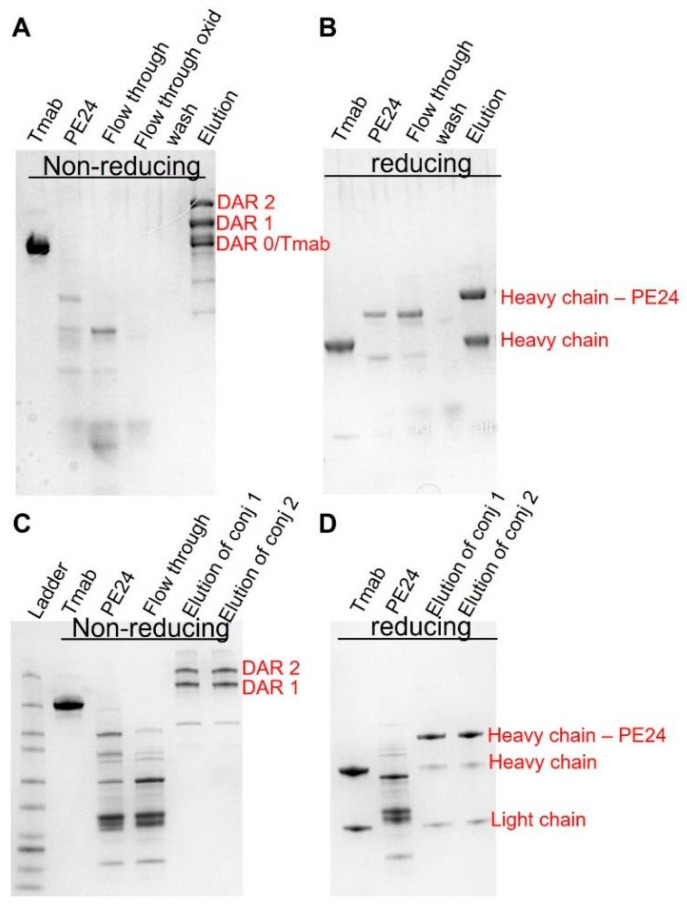
Generation of site-specific trastuzumab-PE. SDS-PAGE analysis of trastuzumab–PE24 conjugates. (**A**) Non-reducing PAGE showing trastuzumab–intein, intein–PE24, flow through from the protein A column, flow through of the oxidized product, wash, and the elutes removing the unconjugated intein–PE24. (**B**) Reducing PAGE showing trastuzumab–intein, intein–PE24, flow through from the protein A column, wash, and the elutes removing the unconjugated intein–PE24. (**C**) Non-reducing PAGE showing trastuzumab–intein, intein–PE24, flow through from the nickel column, the final purified product of conjugate 1 (8 molar excess of Intein-PE) and the final product of conjugate 2 (20 molar excess of Intein-PE). (**D**) Reducing PAGE showing trastuzumab–intein, intein–PE24, the final purified product of conjugate 1 and the final product of conjugate 2.

Given the above simulation results, we postulate that this strategy would have maximal benefit when utilized with antibodies delivering highly potent toxins exhibiting steep concentration–effect relationships and minimal or no bystander activity. To this end, PE24 was conjugated to trastuzumab using site-specific conjugation, enabled by self- splicing split intein. The conjugates were subsequently purified to eliminate unconjugated PE24 through protein A agarose (Figure 4A,B) and to eliminate unconjugated trastuzumab via a Nickel column (Figure 4C,D).

**Figure 5:**
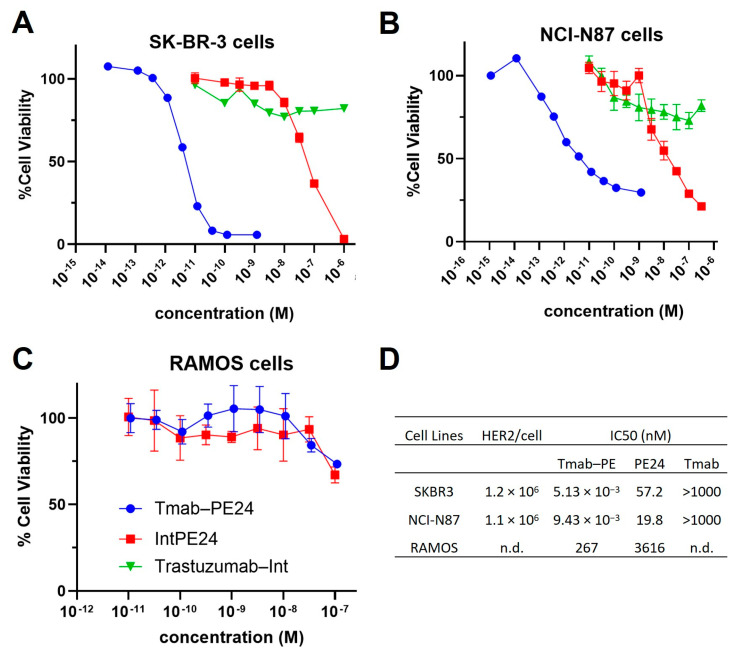
Cytotoxicity of trastuzumab–PE. Shown are the cell viability of (**A**) SKBR3 cells, (**B**) NCI-N87 cells and (**C**) RAMOS cells treated with trastuzumab (blue circles), PE24 (red squares), or trastuzumab–PE24 (green triangles) for 72 h. Points represent the mean of triplicate wells with standard deviation error bars. (**D**) Table listing the IC50s of each construct on different cells.

MTT assays were conducted on various cell lines. The conjugate exhibited potent cytotoxicity, with IC50 values in the low-pM range on high-HER2-expressing cells, such as SKBR3 and N87 cells (Figure 5A,B). The unconjugated PE24 presented IC50s in the medium nanomolar range (Figure 5A,B). The targeting index was between 2000 and approximately 11,000, indicating very high specificity. For RAMOS cells, which do not express HER2, the trastuzumab–PE24 conjugate and PE24 showed high nanomolar IC50s, demonstrating no targeting (Figure 5C). Additionally, trastuzumab–Int exhibited no cytotoxicity effect across all cell lines (Figure 5A–C). The IC50 values of different molecules evaluated on different cell lines are listed in Figure 5D.

### 3.4. Modeling and Simulation for Predicting Ideal Koff of AIDEs

**Figure 6:**
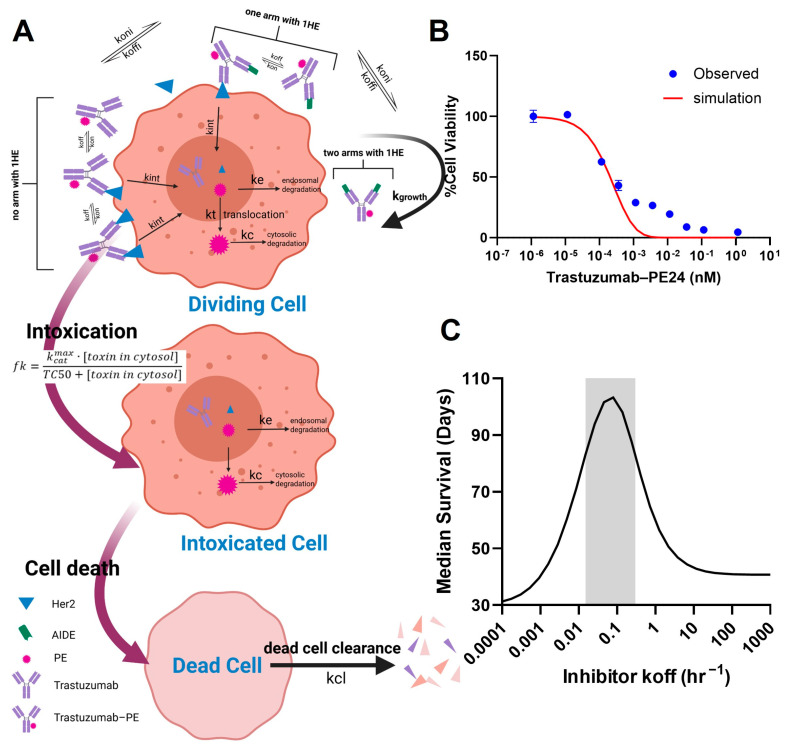
PK/PD modeling and simulation. (**A**) A graphic representation of the PD model structure for the PE intoxication process. (**B**) The observed (blue circles) and PD-model-simulated (red line) cell viability for NCI-N87 cells after treatment with various concentrations of trastuzumab–PE24 for 6 days of incubation. (**C**) The PK/PD model predictions for the relationship between the AIDE koff and the response of NCI-N87 xenografts to a single dose of trastuzumab–PE are shown. The gray shaded region represents an estimated range for a suitable AIDE dissociation rate constant to use in combination of trastuzumab–PE24.

In order to apply the AIDE strategy to improve the intra-tumoral distribution of the high-potency trastuzumab–PE, the key characteristics of AIDEs, such as the optimal dissociation rate (koff), need to be determined. The challenge lies in finding a balance: AIDEs with too low an affinity dissociate too rapidly to significantly improve distribution, while high-affinity AIDEs may outcompete with antibodies for tumor antigens. In the PD model depicted in Figure 6A, we simulated the in vitro viability of NCI-N87 cells subjected to a 6-day incubation with trastuzumab–PE24, and compared this with observed MTT results (Figure 6B). The viability curve was well within range, validating the use of the PD model, which was combined with the sphere PK model for further simulations. The simulations predicted that the coadministration of an AIDE with a koff between 0.015 and 0.3 HR^−1^ could maximize anti-tumor efficacy and extend median survival days when 0.35 mg/kg of trastuzumab–PE24 is administered (Figure 6C). Two anti-trastuzumab AIDEs, LG1 and 1HE, which have koff values within the optimal range [21], were subsequently chosen for the efficacy study.

### 3.5. Effects of AIDEs on Trastuzumab–PE24 Efficacy in Tumor-Bearing Mice

**Figure 7:**
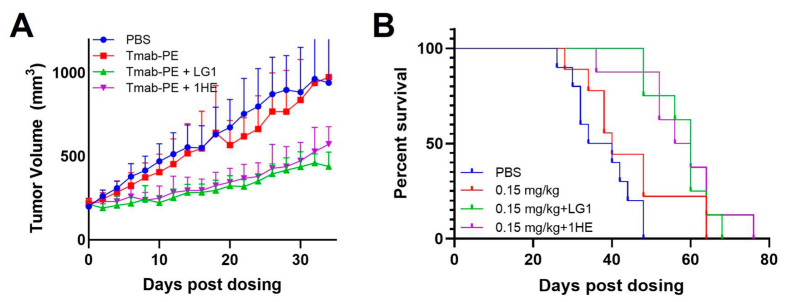
Effects of AIDE co-administration on trastuzumab–PE efficacy in NCI-N87 xenografts. (**A**) Tumor volume over time, (**B**) Kaplan–Meier survival curves of NCI-N87 xenograft-bearing mice following a single intravenous injection of PBS (blue), 0.15 mg/kg trastuzumab–PE (red), trastuzumab–PE+LG1 (green) and trastuzumab–gelonin+1HE (purple).

**Figure 8:**
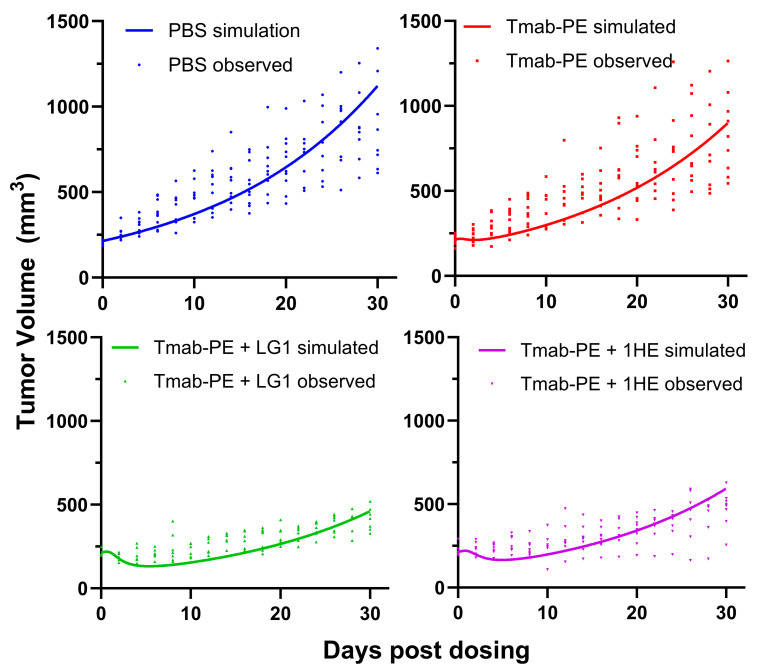
Simulated and observed tumor volumes of NCI-N87 xenograft following a single intravenous injection of PBS (blue), 0.15 mg/kg trastuzumab–PE (red), trastuzumab–PE+LG1 (green) and trastuzumab–gelonin+1HE (purple). The PK/PD model-simulated tumor profiles are shown as solid lines. Solid symbols represent the observed NCI-N87 tumor volumes for individual xenografts.

Both doses of 0.25 mg/kg and 0.6 mg/kg of trastuzumab–PE24 induced body weight loss and caused severe toxicity Eventually, a lower dose of 0.15 mg/kg of trastuzumab–PE24 was applied. The coadministration of LG1 and 1HE with 0.15 mg/kg of trastuzumab–PE24 resulted in reduced tumor volumes, compared to the group receiving trastuzumab–PE24 alone (Figure 7A). Compared to the control group, median survival days extended from 40 days to 60 days (*p* = 0.0002) or 58 days (*p* = 0.0003) with the coadministration of LG1 or 1HE, respectively (Figure 7B). For each group, PK/PD simulations were performed and the results compared with the observed tumor volumes, yielding accurate predictions (Figure 8).

### 3.6. Prediction of Multiple-Dosing Regimen

**Figure 9:**
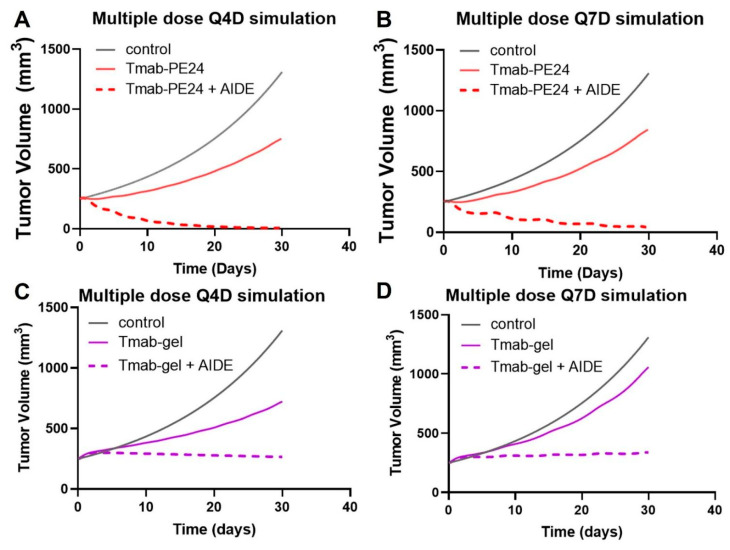
Predicted tumor volumes after multiple doses. The PK/PD model predicts NCI-N87 growth (black line) following (**A**) Q4Dx8 doses of trastuzumab–PE24 (Tmab-PE24, solid red line) or Tmab-PE24 + 10:1 AIDE (dashed red line), (**B**) Q7Dx5 doses of Tmab-PE24 (solid red line) or Tmab-PE24 + 10:1 AIDE (dashed red line), (**C**) Q4Dx8 doses of trastuzumab–gelonin (Tmab-gel, solid purple line) or Tmab-gel + 10:1 AIDE (dashed purple line), and (**D**) Q7Dx5 doses of Tmab-gel (solid purple line) or Tmab-gel + 10:1 AIDE (dashed purple line).

To enhance the benefits of the AIDE strategy while maintaining a tolerated dose, multiple-dosing regimens were simulated. The simulations suggest the low efficacy of the Q4D or Q7D administration of trastuzumab–PE24 alone, while a significant enhancement in anti-tumor effect and tumor regression was predicted with AIDE LG1 co-administration (Figure 9A,B). Conversely, previous simulations of the less potent immunotoxin trastuzumab–gelonin showed that although multiple-dosing regimens of 1HE with trastuzumab–gelonin would enhance efficacy, no tumor regression would occur (Figure 9C,D) [6]. Thus, the AIDE strategy, when applied to more potent toxins, could provide greater benefits.

## 4. Discussion

The BSB has been identified as a limiting determinant of the efficacy of antibody- based therapy for more than 30 years [2,24]. The AIDE approach, first presented by Bordeau et al. in 2021, has emerged as a promising strategy to overcome the BSB. Co-administering the anti-cancer antibody with the anti-trastuzumab anti-idiotypic sdAb (1HE) increased the intra-tumoral penetration of trastuzumab and enhanced the anti-tumor efficacy of trastuzumab-DM1 in NCI-N87 xenografts [5]. In subsequent research, we developed a mechanistic PK/PD model, aiming to predict the effects of AIDEs on the within-tumor exposure of trastuzumab and the therapeutic effect of trastuzumab emtansine (T-DM1). Furthermore, we generated a panel of anti-trastuzumab AIDEs, each with distinct dissociation half-lives ranging from 1.1 h to 107.9 h. This range of options allowed us to tailor the application of the AIDE strategy according to varying conditions, further optimizing its implementation [21]. As our research progressed, we expanded the AIDE strategy to encompass the immunotoxin trastuzumab–gelonin. The coadministration of 1HE with trastuzumab–gelonin resulted in significant increases in the penetration of trastuzumab from the vasculature. This was further underscored by a higher percentage of the tumor area staining positive for trastuzumab–gelonin, an indication of improved intratumoral distribution. Moreover, the combined use of 1HE and trastuzumab–gelonin resulted in enhanced anti-tumor efficacy, characterized by a decreased tumor growth rate and increased median survival [6]. Collectively, these findings substantiate our initial hypothesis that the transient inhibition of antibody–antigen binding, achieved by the coadministration of an AIDE, can bypass the BSB and augment the efficacy of antibody conjugates.

Yet, throughout our investigations, three central questions remained unexplored: What is the impact of AIDEs on the total tumor exposure of mAbs or their conjugates? How do AIDEs affect pharmacokinetic selectivity? And finally, what characteristics should a toxin ideally possess when utilizing the AIDE platform? In this study, we delve into these questions, and further discuss the optimization of the AIDE strategy.

Our sphere model predicted that the coadministration of 1HE would reduce the Cmax of trastuzumab in the tumor, but it would also slow down the terminal slope of the tumor PK. These model predictions were confirmed by our experimental data. We hypothesize that when trastuzumab is administered alone, it tends to bind rapidly to tumor regions closest to the vasculature due to the high affinity binding. Following internalization, trastuzumab undergoes intracellular degradation in the endosome and lysosome. Tumors with high antigen expression, in combination with newly recycled or synthesized antigens, can form a substantial sink for free trastuzumab, consuming considerable amounts in vivo.

When trastuzumab is co-administered with 1HE, 1HE blocks mAb-antigen binding in the perivascular region (layer A). Trastuzumab–1HE complexes then have the potential to diffuse deeper into tumor regions (layers B–E) or return to plasma. Consequently, compared to trastuzumab administered alone, trastuzumab co-administered with 1HE demonstrates a reduction in intracellular degradation and a slower terminal slope of tumor pharmacokinetics, observations validated by both model simulations and experiments.

The diminished tumor Cmax associated with 1HE coadministration can be attributed to the diffusion of trastuzumab–1HE complexes from intratumoral extravasation sites back to the plasma. However, such a decrease in Cmax does not necessarily detract from efficacy, since the concentration of trastuzumab at extravasation sites (represented as “layer A” within the sphere model) is well above concentrations required for efficacy, and is thus superfluous.

We observed a minor change of −2.6% in tumor AUC_0-infinity_ with 1HE coadministration. Although the statistical significance of this difference may not be examined due to the sparse sampling scheme that was employed, we do expect that the very slight reduction in cumulative tumor exposure is biologically insignificant. Indeed, the present efficacy results obtained using trastuzumab–PE24 and our prior efficacy results (obtained using trastuzumab–gelonin and trastuzumab–DM1) suggest that improved intra-tumoral ADC/IT distribution supersedes the impact of the slight reduction in overall tumor AUC, as coadministration with AIDEs was found to significantly increase conjugate efficacy in all cases in our current and prior investigations [5,6,21].

At present, the AUC we have measured accounts for only 60% of AUC_0-infinity_, necessitating further investigations into later time points to foster greater confidence. Beyond the tumor and plasma pharmacokinetics of naked antibodies, future research must also consider the pharmacokinetics of the payloads of ADCs and immunotoxins both with and without AIDE coadministration, since payloads exhibit distinct PK profiles and can dissociate from the antibodies, and mAb-based immunotoxins typically have shorter half-lives compared to naked or deconjugated mAbs [25,26]. These considerations are crucial to advancing our understanding and optimization of the AIDE platform in the future.

We also predict that the AIDE strategy will yield increasing benefits with increasing immunotoxin potency. This prediction is based, in part, on the “overkilling” hypothesis. Briefly, it has been estimated that approximately 0.13 million molecules per cell of internalized trastuzumab–gelonin are necessary to achieve half-maximal cell killing [6]. Consequently, saturating cells with high antigen expression levels (>1 million antigens/cell) may result in approximately 90% of bound trastuzumab–gelonin being “wasted”, as there is a far greater delivery of gelonin than needed for efficient killing [27]. By overcoming the binding site barrier with our AIDE strategy, we predict a more homogenous intratumoral distribution, increasing by up to 10-fold the number of tumor cells receiving lethal doses of toxin. In the case of PE immunotoxins, the delivery of less than 1000 PE molecules per cell is sufficient for cell death [28]. Owing to this high potency, and the predictions of an exponential drop in immunotoxin concentration with increasing distance from extravasation sites, the model predicts that the use of AIDEs with PE immunotoxins will increase by 1000-fold the number of tumor cells receiving lethal exposure. Additionally, it is important to note that the maximal tolerated dose (MTD) decreases with payload potency [29], resulting in the administration of lower doses of antibody conjugates. This reduction in dosage exacerbates the heterogeneity of tumor distribution within tumors (as lower doses yield antigen-saturating concentrations within a lower fraction of the tumor).

To make the trastuzumab–PE24 conjugates, site-specific conjugation was used, a process known to diminish construct heterogeneity. Previous studies have demonstrated that site-specific conjugation could potentially enhance efficacy or tolerability in certain circumstances [30,31,32]. For instance, the cysteine-engineered THIOMAB developed by Genentech, when conjugated with small-molecule payloads, exhibited uniform drug loading. This uniformity was found to significantly improve preclinical in vivo tolerability in comparison to conventional randomly conjugated ADCs [33].

The process of protein conjugation using the split intein M86, as described by Pirzer et al. [20], served as an invaluable reference point for our research. The use of self-splicing intein results in the formation of a new peptide bond. Peptide bonds are generally kinetically stable and resistant to spontaneous hydrolysis under normal physiological conditions. The linker in the final construct between trastuzumab and PE24 is a standard flexible glycine-serine linker, commonly used in immunotoxins to maintain conformational flexibility and functionality.

By adapting this optimized site-specific conjugation method, we were able to consistently, stably, and homogeneously prepare trastuzumab–PE24. Each batch produced featured an average drug–antibody ratio (DAR) of 1.3, further suggesting the reliability of the site-specific conjugation method in yielding a uniform and precise conjugate output.

The dosing range for various types of PE immunotoxins, including scFv-, dsFv-, fab-, albumin binding domain-, and IgG-based immunotoxins, varies extensively in mouse models. It ranges from 0.2 nmol/kg to 12.5 nmol/kg [34,35,36,37,38,39]. This dose range equates to approximately 0.037 mg/kg to 2.3 mg/kg of trastuzumab–PE24, considering an average molecular weight of 184 kDa. Within this broad range, doses between 1.2 nmol/kg and 3.5 nmol/kg of IgG immunotoxins are predominantly employed [34,35,36,37,38,39]. Given this context, we opted for a middle dose of 0.35 mg/kg to perform our simulations. The simulations predicted that an anti-trastuzumab AIDE with the optimal koff could result in a substantial therapeutic advantage, potentially leading to complete tumor regression. Unfortunately, when actual doses of 0.25 and 0.6 mg/kg were administered, they resulted in significant body weight losses and high toxicity. As a result, we ultimately selected a lower dose of 0.15 mg/kg for our in vivo investigations. This dosage was found to induce no observable adverse events and have substantial anti-tumor efficacy. Of note, sensitivity analyses demonstrated that the trastuzumab–PE24 dose, when falling within the range of 0.1–0.35 mg/kg, does not affect the determination of the optimal AIDE koff range. Additionally, the comparison of simulated and observed tumor volumes (Figure 8) demonstrates that the model provides accurate predictions of the effects of 0.15 mg/kg trastuzumab–PE24 with or without the co-administration of model AIDEs (1HE, LG1).

The model has not been validated against external data sets, specifically relating to toxin internalization and cytotoxic thresholds, due to the unavailability of suitable data. The simulation is, however, based entirely on external parameters. We used the trastuzumab internalization rate for the internalization of Tmab-PE [21]. PD parameters including cytosolic translocation rates and cytotoxic thresholds were based on experimental data from literature reports [22,23]. Simulations were evaluated by comparison to our internal data sets.

In the in vivo efficacy study, the results were compared by use of mice receiving AIDE and immunotoxin co-dosing and mice receiving immunotoxin alone (as the comparative control group). Comparisons with other clinically approved ADCs or immunotoxins (e.g., T-DM1, trastuzumab deruxtecan (T-DXd)) might also be useful. The application of AIDE with T-DM1 and trastuzumab–gelonin has been investigated in previous publications [5,6,21]. Investigations of the use of AIDE with T-DXd, as well as other antibody therapeutics, are ongoing in our lab.

PE24 was chosen in this study partly due to its relatively lower immunogenicity than Lumoxiti, shown by LMB-100 in its Phase I clinical studies [18]. Our xenograft work, as is required, employed an immunodeficient mouse model and, consequently, immunogenicity was not assessed (as immunogenicity findings in such models are not expected to be meaningful predictors of clinical immunogenicity).

Immunotoxins harboring PE24 have shown dose-limiting toxicity in clinical use [18]. In the present investigation, all mice received trastuzumab–PE24 at a dose of 0.15 mg/kg, and the two treatment groups (with and without AIDE) exhibited nearly identical body weight profiles, suggesting that AIDE coadministration did not exacerbate systemic toxicity. Given the exploratory nature of this study, we focused on body weight as a general measure of systemic tolerability, and did not perform organ-specific toxicity assessments (e.g., liver enzymes, kidney histology). However, comprehensive toxicity profiling, including serum chemistry and histological analysis of off-target organs, will be pursued in future studies.

Potential mechanisms for the toxicity observed at 0.25 and 0.6 mg/kg doses of trastuzumab–PE24 likely include the non-specific uptake of the immunoconjugates by normal tissues through Fc receptor-mediated endocytosis, or via non-specific endocytosis, leading to unintended cellular internalization and toxin delivery. Due to the high potency of PE24, even low levels of trastuzumab–PE24 endocytosis may contribute to systemic toxicity and lead to a narrow therapeutic index. From a clinical development perspective, our findings emphasize the importance of careful dose selection for PE-based immunotoxins. Notably, the coadministration of AIDEs enabled the attainment of substantial antitumor efficacy at a low dose (0.15 mg/kg), improving the therapeutic index of trastuzumab–PE24.

Therapeutic antibodies like trastuzumab can often activate immune responses and mediate antibody-dependent cellular toxicity, a function that AIDEs are unlikely to alter. AIDEs bind to the idiotopes of the antibody in the variable regions on the Fab fragments, and AIDEs are not expected to bind to or interfere with the Fc domains that are responsible for the activation of antibody-associated immune responses. Of note, our AIDE candidates are single-domain antibodies with a small size of only ~13 kDa. As such, we expect that the use of AIDEs will not sterically hinder Fc function or influence the ability of anti-cancer antibodies to activate immune responses.

The potential impact of antibody therapeutics and AIDE co-administration on cells with low antigen expression is an interesting topic worth discussion. Two cell types may be considered—healthy cells with low antigen expression and tumor cells with low antigen expression. Our strategy of the co-administration of AIDEs with ADCs and ITs is designed to transiently block antibody–antigen binding, and therefore target-mediated disposition. AIDE treatment is anticipated to shift antibody exposure from more accessible areas (e.g., healthy tissues with better blood perfusion, perivascular regions in tumor) to less accessible areas (e.g., deeper regions in solid tumors). Therefore, for healthy tissues, we anticipate that the use of AIDEs will decrease the biological effects of antibody conjugates, as healthy tissues are typically well-perfused when compared to solid tumors, and healthy tissues are typically associated with fewer barriers to antibody distribution relative to solid tumors. Indeed, AIDE co-administration with trastuzumab–PD24 led to no observable increase in systemic toxicity in the present efficacy study (i.e., assessed by body weight loss). In addition, the AIDE approach allowed us to achieve therapeutic efficacy at lower doses of trastuzumab–PE24, which could potentially allow reduced systemic toxicity if applied in clinical oncology.

The AIDE strategy is designed to mitigate the BSB, which is more pronounced for high-affinity antibodies in tumors with high antigen expression, where antibodies bind rapidly and are retained in perivascular regions. In tumors with low antigen expression, the BSB is likely less significant, and the predicted benefit of AIDE coadministration on antibody distribution and efficacy is likely to be less substantial.

The anticipated effects of AIDEs on the disposition and toxicity of ADCs or ITs in healthy tissues and in tumors with low antigen expression require further investigation, perhaps including investigations in transgenic mouse models bearing tumor xenografts, where it may be possible to consider substantial ranges of antigen expression, tissue perfusion, intra-tissue/tumor diffusivity, and sensitivity to payloads.

## 5. Conclusions

In summary, this study demonstrated that anti-trastuzumab anti-idiotypic distribution enhancers (AIDEs), which were previously shown to improve the intra-tumoral distribution of trastuzumab, did not have substantial, negative impacts on overall tumor exposure (i.e., tumor AUC) or on the pharmacokinetic selectivity of trastuzumab. Model AIDEs, 1HE and LG1, dramatically increased the anti-cancer efficacy of trastuzumab–PE24 in NCI-N87 xenograft-bearing mice. The mathematical modeling and simulations accurately predicted anti-tumor efficacy, and also predict that multiple doses of AIDE coadministration with trastuzumab–PE24 could result in complete tumor regression. Our modeling work predicts that the benefits of AIDE coadministration with antibody conjugates will increase with increasing payload potency.

## Data Availability

Dataset available on request from the authors.
